# Case report: Unveiling a less severe congenital nephrotic syndrome in a Rapa Nui patient with a *NPHS1* Maori founder variant

**DOI:** 10.3389/fneph.2024.1379061

**Published:** 2024-05-14

**Authors:** Paola Krall, Angélica Rojo, Anita Plaza, Sofia Canals, María Luisa Ceballos, Francisco Cano, José Luis Guerrero

**Affiliations:** ^1^ Instituto de Medicina, Facultad de Medicina, Universidad Austral de Chile, Valdivia, Chile; ^2^ Departamento de Pediatría y Cirugía Infantil Oriente, Facultad de Medicina, Universidad de Chile, Santiago de Chile, Chile; ^3^ Unidad de Nefrología, Diálisis y Trasplante Renal, Hospital Luis Calvo Mackenna, Santiago de Chile, Chile; ^4^ Unidad de Lactantes, Hospital Luis Calvo Mackenna, Santiago de Chile, Chile; ^5^ Programa de Telesalud, Hospital Luis Calvo Mackenna, Santiago de Chile, Chile

**Keywords:** congenital nephrotic syndrome, NPHS1, kidney survival, Maori founder variant, Rapa Nui (Easter Island)

## Abstract

**Background:**

Congenital nephrotic syndrome (CNS) is a severe kidney disorder characterized by edema, massive proteinuria, and hypoalbuminemia that manifests *in utero* or within three months after birth. CNS affects 1-3 per 100,000 children, primarily associated with genetic variants and occasionally with infections. Genetic analysis is the first-line method for diagnosis. The most common founder variants have been identified in European populations, often resulting in end-stage kidney disease by 1-2 years of age.

**Case-diagnosis/treatment:**

A female full-term neonate, without prenatal signs of kidney disease, was admitted to Rapa Nui (Eastern Island) Hospital at the age of 2 months due to bronchial obstruction. She presented fever, oliguria, edema, urine protein-to-creatinine ratio (UPCR) 433.33, and hypoalbuminemia (0.9 g/dL). She was transferred to a mainland Chilean hospital following CNS diagnosis. Viral screening detected cytomegalovirus (CMV) positivity in both blood and urine. A kidney biopsy revealed interstitial nephritis and diffuse podocyte damage and the tissue PCR resulted negative for CMV. Interviews with the parents revealed consanguinity, suggestive of hereditary CNS. Genetic analysis identified the Maori founder variant, *NPHS1* c.2131C>A (p.R711S), in homozygosis. The patient received albumin infusions and antiviral therapy, being discharged when she was 5 months old, with improved laboratory parameters evidenced by UPCR 28.55, albumin 2.5 g/dL, and cholesterol 190 mg/dL. Subsequent clinical monitoring was conducted through virtual and in-person consultations. At her last follow-up at 4 years 2 months old, she presented UPCR 16.1, albumin 3.3 g/dl and cholesterol 220 mg/dL, maintaining normal kidney function and adequate growth.

**Conclusions:**

To our knowledge, this represents the first case of CNS in Chile carrying a *NPHS1* variant associated with prolonged kidney survival. As described in the Maori population, the patient exhibited a less severe clinical course compared to classical *NPHS1* patients. Genetic testing for the Maori founder variant in CNS patients related to the New Zealand population, could impact management decisions and potentially prevent the need for nephrectomies.

## Introduction

Congenital nephrotic syndrome (CNS) is a severe kidney disease that is present at birth or manifests within the first three months of life with clinical signs of edema, massive proteinuria and hypoalbuminemia (1). CNS affects 1-3 per 100,000 newborns, turning this kidney disease into a rare condition, although a higher frequency in specific populations has been described reaching up to 1 in 8,000 in Finland (2). CNS is associated with genetic variants encoding proteins that are expressed in the glomerular filtration barrier, including those found in the slit diaphragm. These genes include nephrin (*NPHS1*), podocin (*NPHS2*), Wilms tumor 1 (*WT1*), Phospholipase C epsilon 1 (*PLCE1*) and Laminin β2 chain (*LAMB2*) (3). However, rare cases of viral-associated glomerulopathies have been described to trigger CNS, reinforcing the need for virus screening (4).

Most of the genetic variants in CNS patients have been detected in *NPHS1*, exhibiting an autosomal recessive inheritance pattern due to homozygous or compound heterozygous combinations (5, 6). As of the current date, over 1200 variants have been documented in *NPHS1*, with approximately 25% classified as either pathogenic or likely pathogenic. The most common *NPHS1* variants, Fin-major and Fin-minor, were identified in Finland as result of a founder effect (7). However, both have been observed in other geographic populations, indicative of a broader involvement of these variants in hereditary CNS (8).

CNS associated with *NPHS1* is a form of steroid-resistant nephrotic syndrome but is treated differently in comparison with patients of infant childhood. The primary objective during the initial evolution of CNS is to manage edema and prevent severe complications such as infections, thrombosis, and/or failure to thrive, which might contribute to morbidity. The clinical course of CNS associated with *NPHS1* usually leads to the requirement of nephrectomy and kidney replacement therapy at 1-2 years of age. Histopathological findings are commonly nonspecific, exhibiting mesangial hypercellularity, glomerulosclerosis, dilated proximal tubules, and diffuse foot process effacement. For this reason, routine kidney biopsy might be replaced by non-invasive molecular diagnosis approaches such as genetic testing (9).

Genetic analysis has become the primary method, offering high detection rates and is strongly recommended to confirm CNS diagnosis, aiding in management, and establishing prognosis (9). If a *WT1* variant is identified, there is a high likelihood to consider a native nephrectomy prior to transplantation (10). Nevertheless, the consideration of aggressive treatment for CNS needs to be analyzed on a case-by-case basis and might benefit of genetic testing, as some patients might require a nephrectomy before transplantation while others have a delayed progression and do not reach end-stage kidney disease by the age of 2 years (11, 12).

In this study, we present a female patient born from a consanguineous couple who was diagnosed with CNS within the first three months of life, representing the first confirmed case from Rapa Nui carrying a known *NPHS1* founder variant associated with prolonged kidney survival.

## Case report

A female newborn patient (gestational age 37 weeks, birth weight 2.82 kg) without prenatal evidence of kidney disease was admitted at the age of 2 months at Hanga Roa Hospital in Easter Island (insular Chilean territory) due to bronchial obstruction that was treated with oral steroids and bronchodilator therapy. Shortly after, she developed fever (38°C) with significant shortness of breath that was treated with oxygen supplementation, but in 24 hours she presented decreased diuresis, abdominal distension and swelling in the lower limbs. Chest radiography did not evidence anomalies. Laboratory findings revealed hypoalbuminemia (0.9 g/dL) and proteinuria as urine protein to creatinine ratio (UPCR) 433.33, consistent with a diagnosis of CNS. Her serum creatinine levels were within normal range (0.2 mg/dL). Subsequent treatment involved 20% albumin over 12-hour infusions (2 g/kg/day) to support intravascular volume and reduce extravascular fluid retention when symptomatic hypovolemia was suspected and red blood cells transfusion due to anemia, as evidenced by hemoglobin of 5.9 g/dL.

She was transferred to a major pediatric hospital in mainland Chile at 2 months and 15 days of life. At the initial assessment, the physical examination confirmed persistence of edema of the eyelids and lower limbs. Laboratory exams upon admission showed hypoalbuminemia (1.5 g/dL), UPCR 156.88, hypocalcemia (6.3 mg/dL) and hypomagnesemia (1.1 mg/dL). The 24-hour urine protein test yielded 8.9 grams (1374 mg/m^2^/hr). Ultrasound showed enlarged kidneys, diffuse echogenic parenchyma and limited corticomedullary differentiation.

The treatment regimen consisted of initiation of captopril at a dose of 1.5 mg/kg per administration, given every 8 hours, resulting in a cumulative daily dose of 4.5 mg/kg. Furosemide was administered at 0.5 mg/kg following each albumin infusion or in the presence of severe edema. Furthermore, the patient received a daily oral dose of 4000 IU (25 mcg/kg/day) of vitamin D3 (cholecalciferol) to maintain target serum levels between 30 to 50 ng/mL. In case of low plasma total IgG levels, 450 mg/kg of intravenous normal human immunoglobulins were administered. Additionally, a daily dose of 25 mcg of levothyroxine was provided (equivalent to 100 mcg/m²/day).

**Figure 1:**
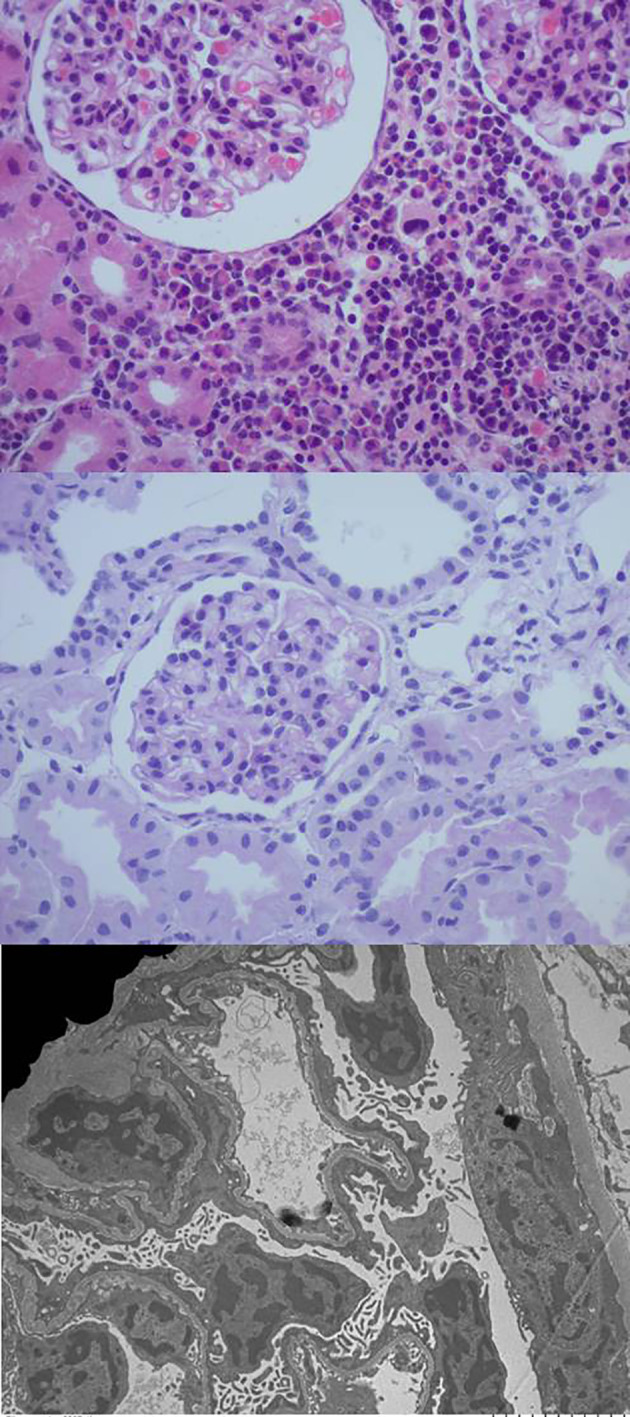
The optical microscopy of the kidney biopsy reveals preserved glomeruli with mild mesangial hypercellularity and a mixed interstitial inflammatory infiltrate, while electron microscopy images highlight marked diffuse podocyte damage.

Screening for viral infections yielded a positive result for cytomegalovirus (CMV) in both blood (1748 copies/mL) and urine (59250 copies/mL). Serologic analysis for human immunodeficiency virus, syphilis and toxoplasmosis was negative. Intravenous ganciclovir treatment was given at a dose of 6 mg/kg every 12 hours for 28 days, followed by oral valganciclovir at a dose of 15 mg/kg every 12 hours for the following 6 weeks. After this treatment, the CMV viral load was negative. A kidney biopsy revealed interstitial nephritis and diffuse podocyte damage and the tissue PCR resulted negative for CMV (**Figure 1**).

**Figure 2:**
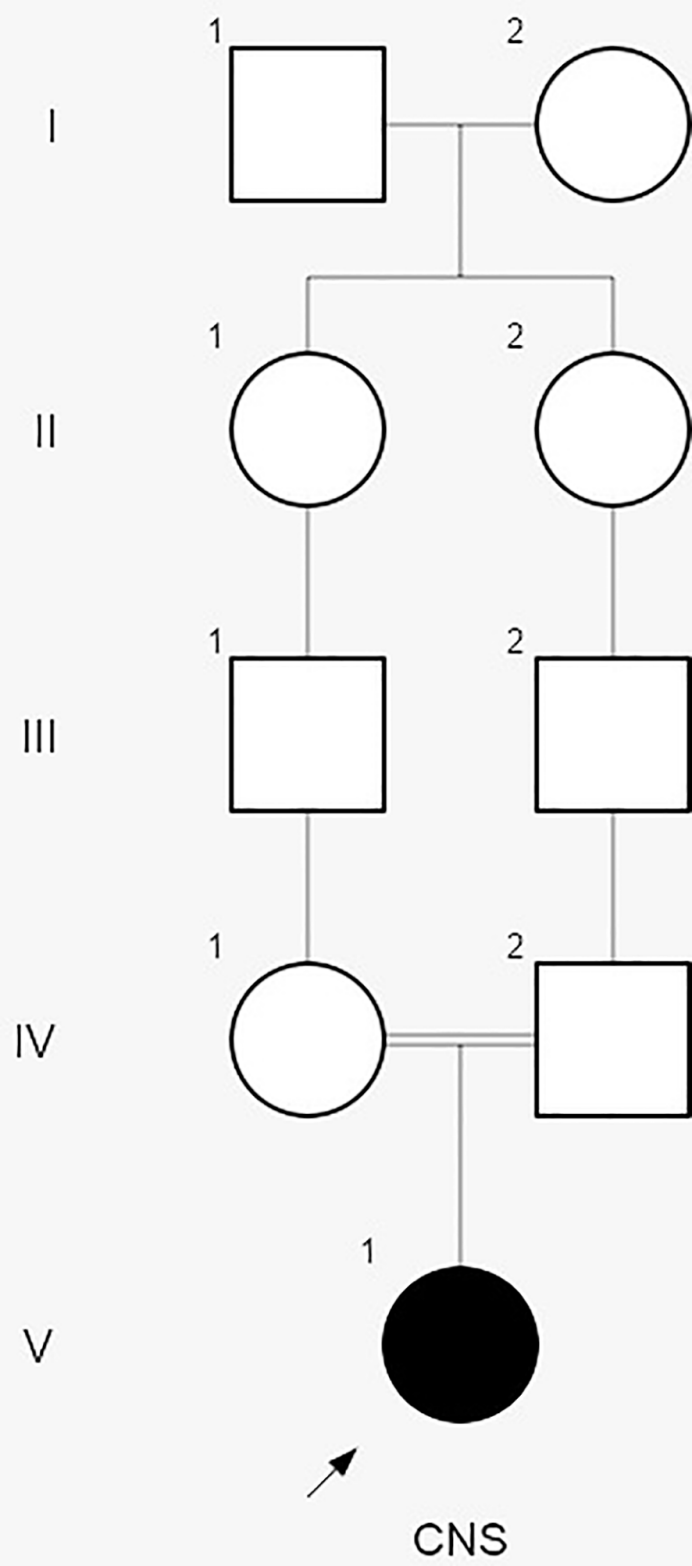
The pedigree chart of the Rapa Nui patient diagnosed with congenital nephrotic syndrome (CNS). During the interview with the parents, no family history of kidney disease was reported. However, they disclosed that the patient´s great grandmothers were sisters, suggesting a potential hereditary etiology for CNS.

**Figure 3:**
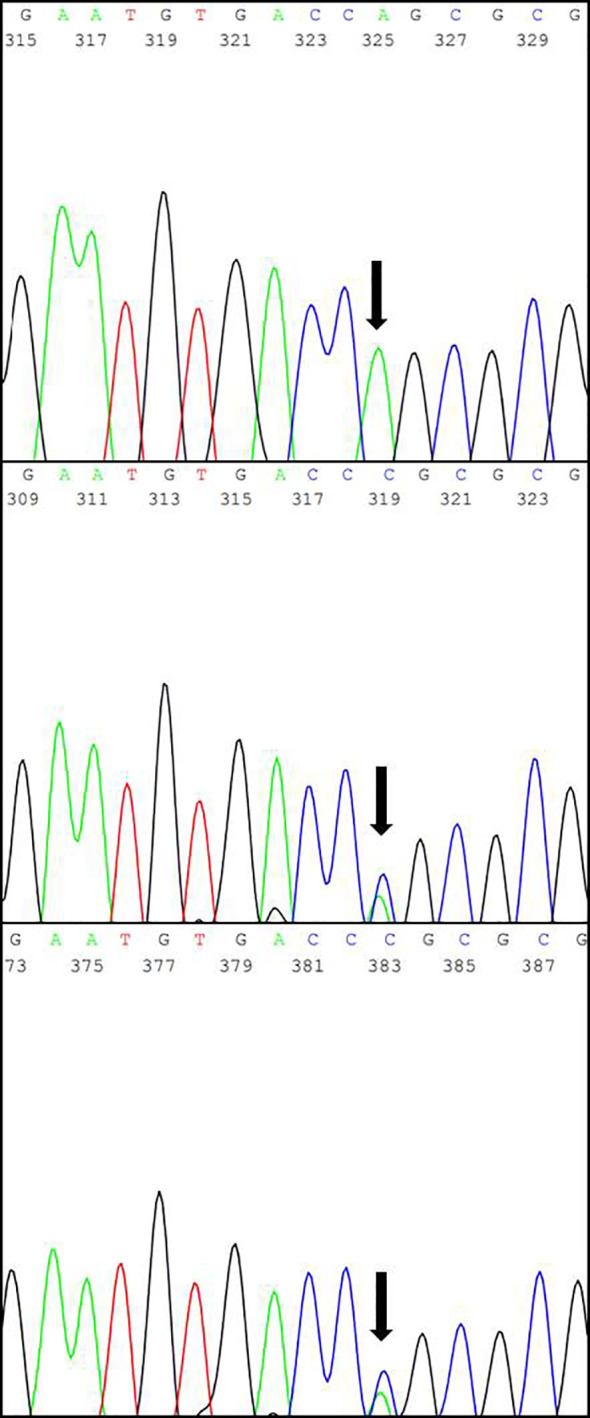
The results of Sanger sequencing showing the specific replacement of a cytosine (C) by an adenine (A) within exon 16 in the *NPHS1* gene (marked with black arrow), predicted to cause the missense variant R711S. Top: Patient; Center: Father; Bottom: Mother.

Subsequent interviews with the parents revealed a consanguineous relationship, suggestive of hereditary CNS (**Figure 2**). Genetic analysis conducted by the Laboratory of Nephrology at the Universidad Austral de Chile identified the variant *NPHS1* c.2131C>A (p.R711S) in homozygosis, described as a Maori founder variant, and present in different population databases (Human Gene Mutation Database, gnomAD, ClinVar, dbSNP). The amino acid substitution is located in a highly conserved region across vertebrate species. Both parents were confirmed to be heterozygous carriers of the *NPHS1* R711S variant (**Figure 3**), prompting genetic counseling about the recurrence in forthcoming gestations considering that the case involved the birth of their first child.

The patient received 20% albumin infusions (2 g/kg/day) and continued antiviral therapy, observing progressive improvement in her general health status. She was discharged when she was 5 months old with favorable laboratory parameters: UPCR 28.55, albumin 2.5 mg/dl and cholesterol 190 mg/dl with indication of treatment with captopril and vitamin D. Clinical follow-up was conducted via telemedicine between Rapa Nui and a nephrologist on mainland Chile during travel restrictions imposed by the SARS-CoV-2 pandemic. This continued until the age of 3 years when in-person visits replaced remote clinical monitoring. At her last visit at 4 years 2 months old, her UPCR was 16.1, albumin 3.3 mg/dL, creatinine 0.27 mg/dL and cholesterol 220 mg/dL (**Table 1**). She maintained normal kidney function, without swelling, normal blood pressure below the 95th percentile for age, gender, and height, and a normal nutritional status (weight-for-age z-score: +0.41, height-for-age z-score: +1.3, body mass index: 14.7 kg/m², body mass index z-score: -0.49). Her body weight was 17.2 kilograms, and she was receiving captopril 12.5 mg three times a day, spironolactone 7.5 mg twice a day and vitamin D 2000 IU once a day.

**Table 1:** Laboratory results by age during the follow-up.

Age	2 months	3 months	4 months	5 months	6 months	8 months	10 months	1 year	3 years	4 years
UPCR	433.33	62	46	28.55	28	9.4	5.9	0.96	6.18	16.1
Serum albumin (g/dL)	0.9	1.7	2.1	2.5	2.6	3.1	3.4	3.4	3.3	3.3
Serum creatinine (mg/dL)	0.2	0.15	0.15	0.15	0.15	0.15	0.22	0.22	0.24	0.27
Serum cholesterol (mg/dL)	127	213	220	190	170	171	170	184	300	220

## Discussion

Diagnosing patients with CNS involves many difficulties, given their susceptibility to hemodynamic compromise, infections, thrombosis, impaired growth and kidney failure. It is important to address and treat potential complications to reduce their morbidity and mortality. Comprehensive CNS care involves the use of renin-angiotensin system inhibitors, diuretics, anticoagulation, and infection prophylaxis, to maintain intravascular euvolemia, ensure proper nutrition, and preserve both central and peripheral vessels. Routine nephrectomies are not recommended, but they may be considered in cases of severe complications despite optimal conservative treatment (9).

In this article, we describe a newborn patient exhibiting CNS with homozygous status of a *NPHS1* variant and CMV infection. Congenital infections, in particular CMV, *Treponema pallidum* (syphilis), and occasionally *Toxoplasma gondii*, along with other pathogens, have been linked to rare occurrences of CNS. Although the presence of CMV and cytomegalic inclusions has been observed in the renal tubular epithelial cells in CMV-associated glomerulopathies, the causative role of this virus in the case of glomerulonephritis and nephrotic syndrome remain subjects of ongoing debate (13).

The patient’s treatment regimen encompassed the administration of albumin over 12 hour infusions when symptomatic hypovolemia was suspected and red blood cell transfusion to address anemia, alongside antiviral therapy during the postnatal period. Hypocalcemia and hypomagnesemia can be observed in patients with nephrotic syndrome due to proteinuria and tubular dysfunction. Furthermore, hypoalbuminemia can reduce the binding of calcium and magnesium in the blood, worsening their loss. Intravenous and oral calcium and magnesium were administered to the patient until their plasma levels were restored to normal.

Discharge occurred at 5 months of age, marked by favorable laboratory parameters. A therapeutic plan was instituted involving vitamin D supplementation, immunoglobulin therapy, levothyroxine and captopril. At her last visit, the patient was 4 years and 2 months old and maintained normal kidney function, exhibited no signs of swelling, normal blood pressure, and sustained nutritional well-being although she continued to exhibit proteinuria through captopril, spironolactone and vitamin D.

The primary goal is to achieve optimal nutrition and growth, mitigate complications, minimize their impact, and attain adequate weight/height through conservative measures, thereby eliminating the need for nephrectomies (11). This has remained the prevailing situation until now in our patient.

Given the predominant association of CNS with *NPHS1*, genetic analysis in the patient was specifically targeted towards this gene. The concern regarding her prognosis stemmed from the fact that the majority of reported CNS cases with *NPHS1* variants developed a severe clinical course involving Caucasian cohorts. However, in 2013, a retrospective study involving 35 patients with CNS reported a subgroup of patients in New Zealand carrying a previously unknown *NPHS1* variant, R711S, which was interpreted as probably disease-causing (14). The variant was confirmed in homozygosis in 10 individuals. All of them were identified as Maori or Maori-descendent and they appeared to show a better response to pharmacological treatment than Caucasians becoming independent of albumin infusion during their second year of life. Their mean kidney survival was 12.4 years (range: 1 - 37 years) with optimal outcomes after transplantation.

Concerning the inhabitants of islands in New Zealand, as well as those of Rapa Nui, the genetic evidence confirms the link between these populations (15). It is recognized that geographically and culturally isolated regions often exhibit higher rates of consanguinity. This contributes to the occurrence of rare autosomal recessive phenotypes related to alleles carried by historic settlers and subsequent migrants. To our knowledge, the patient we describe here is the first reported case outside New Zealand with this unique genotype as a homozygous carrier of the Maori founder variant. This is more likely to be explained by the inheritance of the variant over many generations, supported by the historical relationship between Rapa Nui and the Maoris. Herein, we describe her clinical course for at least 4 years, evidencing a less severe phenotype similar to the one reported in Maori CNS patients.

In summary, a comprehensive evaluation of CNS is necessary to enhance our understanding of its pathogenesis, involving genetic, viral studies and biopsy. Molecular testing is crucial for determining both short-term and long-term prognosis, as well as formulating an effective treatment plan.

## Data availability statement

The datasets for this article are not publicly available due to concerns regarding participant/patient anonymity. Requests to access the datasets should be directed to the corresponding author.

## Ethics statement

The studies involving humans were approved by Comité Ético Científico Servicio de Salud Valdivia. The studies were conducted in accordance with the local legislation and institutional requirements. Written informed consent for participation in this study was provided by the participants’ legal guardians/next of kin. Written informed consent was obtained from the individual(s) for the publication of any potentially identifiable images or data included in this article.

## Author contributions

PK: Conceptualization, Formal Analysis, Funding acquisition, Methodology, Supervision, Writing – original draft, Writing – review & editing, Data curation, Project administration, Resources, Validation. AR: Data curation, Writing – review & editing. AP: Data curation, Methodology, Writing – review & editing. SC: Data curation, Writing – review & editing. MC: Data curation, Writing – review & editing. FC: Data curation, Writing – review & editing. JG: Conceptualization, Data curation, Formal Analysis, Supervision, Writing – original draft, Writing – review & editing.
