# Corrigendum: Case report: Unveiling a less severe congenital nephrotic syndrome in a Rapa Nui patient with a *NPHS1* Maori founder variant

**DOI:** 10.3389/fneph.2024.1454138

**Published:** 2024-07-03

**Authors:** Paola Krall, Angélica Rojo, Anita Plaza, Sofia Canals, María Luisa Ceballos, Francisco Cano, José Luis Guerrero

**Affiliations:** ^1^ Instituto de Medicina, Facultad de Medicina, Universidad Austral de Chile, Valdivia, Chile; ^2^ Departamento de Pediatría y Cirugía Infantil Oriente, Facultad de Medicina, Universidad de Chile, Santiago de Chile, Chile; ^3^ Unidad de Nefrología, Diálisis y Trasplante Renal, Hospital Luis Calvo Mackenna, Santiago de Chile, Chile; ^4^ Unidad de Lactantes, Hospital Luis Calvo Mackenna, Santiago de Chile, Chile; ^5^ Programa de Telesalud, Hospital Luis Calvo Mackenna, Santiago de Chile, Chile

**Keywords:** congenital nephrotic syndrome, NPHS1, kidney survival, Maori founder variant, Rapa Nui (Easter Island)

In the published article, there was an error in the Funding statement. The funding statement for the article was displayed with the recognition of FONDECYT #111-40242. The correct statement is FONDECYT #111-40242 and INGE 210028 from the Vicerrectoría de Investigación y Desarrollo, Universidad de Chile. The correct Funding statement appears below.

**Funding**

The author(s) declare financial support was received for the research, authorship, and/or publication of this article. The genetic analysis was funded by the FONDECYT #111-40242. The publication of this article was funded by INGE 210028 from the Vicerrectoría de Investigación y Desarrollo, Universidad de Chile.

The authors apologize for this error and state that this does not change the scientific conclusions of the article in any way. The original article has been updated.

